# Antibacterial Borosilicate Glass and Glass Ceramic Materials Doped with ZnO for Usage in the Pharmaceutical Industry

**DOI:** 10.1021/acsomega.3c00720

**Published:** 2023-05-18

**Authors:** Barış Demirel, Melek Erol Taygun

**Affiliations:** † Department of Chemical Engineering, Istanbul Technical University, Maslak, Istanbul 34469, Turkey; ‡ Sisecam Science Technology and Design Center, Gebze, Kocaeli 41400, Turkey

## Abstract

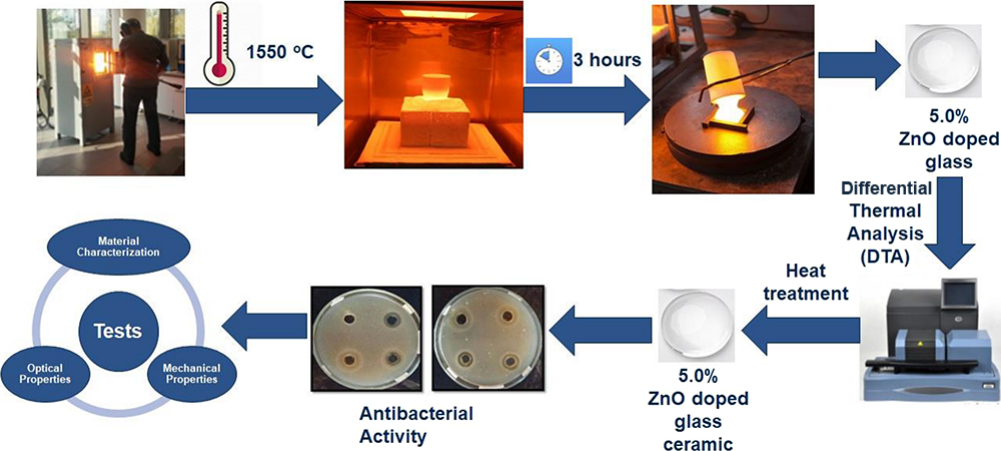

The aim of this study is producing and characterizing borosilicate glass and glass ceramic materials with enhanced antibacterial properties by using the conventional melting method. First of all, borosilicate glass doped with ZnO was obtained and after that the crystallization temperature was detected by using differential thermal analysis for the production of borosilicate glass ceramic doped with ZnO. The antibacterial and leaching tests showed that the glass and glass ceramic doped with 5% ZnO were suitable samples according to test results. Physical, thermal, and mechanical properties of the glass and glass ceramic doped with 5% ZnO were also determined. Overall results indicated that the obtained antibacterial borosilicate glass could be a remarkable product for the pharmaceutical industry, especially for usage in drug packaging.

## 1 Introduction

Glass has a proven contribution to human civilization reaching modern times. Important glassy materials have a history of 5000 years.^1^ Glass and glass ceramic materials have a wide range of use and are essential in modern life. Glass is commonly used in important areas of human life like food and beverage, glassware, medicine, etc.^2−4^ Glass ceramic has also a significant area of usage; some examples are in military application, dentistry, and household appliances.^5−7^ With increased investments in the glass industry, more research studies have performed studies to discover the opportunities to improve the effect of glass and glass ceramics on human health.^8−11^

It is known that infectious diseases lead to changes in human life.^12^ Different types of microorganisms (viruses, bacterium, fungi, etc.) cause infectious diseases, which influence human life.^13^ Such diseases have common undesirable economic effects besides their mortality and morbidity.^12^ Although globalization is a must in the current economic environment, it leads to spread of pathogens worldwide.^12^ Bacterial infections are treated with different types of antibiotics, which are becoming challenging day by day, because of increased resistance of microorganisms to antibiotics.^14^ Therefore, the glass industry accelerated the investigations on glass materials with enhanced antibacterial properties.^15−20^ Many ingredients like copper, silver, zinc, titanium oxide, etc. have been used for such purpose.^13,15−26^

Antibacterial glasses have many areas of usage like glassware, packaging, or health sector. Glasses with antibacterial properties are used in hematology, oncology, and burn units in the hospitals and their importance is increasing day by day. Additionally, an increase in the usage of borosilicate glass materials has been observed during pandemic for drug packaging, especially for the packaging of the vaccines.^27−29^ Also, higher demand on long-term storage of food and beverages shows that antibacterial glasses become more important in the food industry. The size of the global antibacterial glass market is expected to be 358.5 million dollars in 2027.^30^

U.S. Food and Drug Administration (21CFR182.8991) defines zinc oxide to be “generally recognized as safe” (GRAS). Zinc oxide is very commonly used in packaging of food for preservation and against spoilage.^31^ It is also known that zinc oxide has antibacterial properties, effective in both major pathogens and different microorganisms.^14^

Antibacterial glass and glass ceramic studies in the literature generally focus on bioglass and bioglass–ceramics.^32−35^ In addition, the antibacterial properties of industrial glass and glass ceramics are obtained by using different methods such as coating or ion exchange.^8,19,20,36−40^

This study aims to investigate and produce the first commercial borosilicate glass and glass ceramic materials with enhanced antibacterial properties obtained by the classical melting method for the glass market. Detailed characterization methods were also performed on the obtained samples to determine the usage areas of antibacterial borosilicate glass and glass ceramic materials.

## 2 Experimental Study

### 2.1 Glass Composition and Melting

A typical borosilicate glass (reference sample as nonbacterial one) is composed of 78.69% SiO_2_, 2.50% Al_2_O_3_, 0.01% Fe_2_O_3_, 0.04% TiO_2_, 0.02% CaO, 5.05% Na_2_O, 0.04% K_2_O, and 13.65% B_2_O_3_ in wt %. Antibacterial properties of medical packaging materials became more critical especially during the pandemic period. Soda-lime glass doped with 5.0% zinc oxide was shown in a previous study to have an antibacterial effect on *Escherichia coli* (*E. coli*).^41^ Therefore, in this study, zinc oxide (ZnO, 99.0% purity) in 5.0% amount was added as an antibacterial component to borosilicate glass batch by reducing the amounts of SiO_2_, CaO, and MgO. Zinc oxide was added in borosilicate glass and glass ceramic composition in this study. The antibacterial borosilicate glass has a composition of 73.69% SiO_2_, 2.50% Al_2_O_3_, 0.01% Fe_2_O_3_, 0.04% TiO_2_, 0.02% CaO, 5.05% Na_2_O, 0.04% K_2_O, 13.65% B_2_O_3_, and 5.00% ZnO in wt %.

Hereafter, a glass batch of about 120 g, prepared by weighing according to a suitable recipe from raw materials and auxiliary substances to give the oxides in the structure of the glass, containing approximately 5.0% zinc oxide, was prepared. The glass batch was melted in an electric furnace at 1550 °C for 3 h in an air atmosphere with a quartz crucible (100% purity). Annealing of the glasses was performed at 570 °C for 1 h followed by slow cooling to room temperature to remove thermal residual stress.

Finally, the crystallization process was applied to borosilicate glass sample doped with 5.0% ZnO at 800 °C for 5 h at a heating rate of 10 °C/min to obtain a glass ceramic structure.

### 2.2 Characterization of Antibacterial Materials

TA SDT Q600 Model thermal analyzer was used for differential thermal analysis (DTA) scans of annealed glass samples. DTA experiments were performed by heating 20 mg glass samples in a Pt-crucible and using Al_2_O_3_ as a reference material in the temperature range between 25 and 1100 °C.

Antibacterial properties of the obtained samples as gram negative were evaluated by using a solid medium. The International Organization for Standardization (ISO) 22196:2011 standard method (Measurement of antibacterial activity on plastics and other nonporous surfaces) was followed for the measurement of antibacterial activity of samples against *E. coli* (American Type Culture Collection (ATCC) 25922). Glass and glass ceramic samples were inoculated with approximately 1 × 10^5^ bacteria/mL of inoculum and coated with a sterile film, after cleaning control and test samples (50 mm × 50 mm) and sterilization of both sides for an hour under ultraviolet (UV) irradiation (in total 2 h). The films were removed from the control and test samples after 24 h of incubation, and the *E. coli* amount was counted. Each analysis was performed in quadruplicate.

Toxicity Characteristic Leaching Procedure (TCLP),^42^ which has acceptable limits and is relatively simple, was used for the investigation of the borosilicate glass and glass ceramic samples’ zinc leachability. A leaching solution (extraction fluid) was used in the TCLP experiments. The glass and glass ceramic samples were put in a conical flask after they were crushed manually under 9.5 mm. The liquid-to-solid ratio was kept in 20 (L/S = 20) by adding the leaching solution. The flask is tightly closed and stored at 25 °C for 18 h. A filter of 0.6–0.8 μm was used for filtering the final solutions, and ICP-OES was used to determine the concentration of zinc ions in the leachate.^43^

Amorphous structure of the glasses and crystalline structure of the glass ceramics were observed by using an X-ray diffraction (XRD) analyzer (RIGAKU Smartlab, 100 mA, 30 kV, scan range:10–90°, step size: 0.01°).

Raman analysis was performed using a RENISHAW inVia spectrometer with 532 nm excitation laser, 50% laser power, 10 s acquisition time, and ×50 objective lens. The Raman curves are the average of analyzing each sample at three different points.

Scanning electron microscopy (SEM) analysis was performed using a QUANTA FEG 250 Field Emission on glass–ceramic sample to be able to detect the crystalline structure. Before the observation, the surface of the sample was sputter-coated (SC7620 sputter coater, Quorum Technologies Ltd., United Kingdom) with platinum for 120 s.

Energy levels of zinc oxide are critical for the antibacterial property of glass and glass ceramics; this was determined by using X-ray photoelectron spectroscopy (XPS) analysis with a Thermo Scientific K-Alpha spectrometer. An aluminum anode (Al Kα = 1468.3 eV) at an electron take-off angle of 90° on the spectrometer was placed between the sample surface and the axis of the analyzer lens. Charging was avoided by using a flood gun. Accelerated Ar ions at 3000 eV for 30 s were used to clean the top surface from any organic impurities. The spectra were recorded in an Avantage 5.9 data system.

A PE Lambda 900/950 ultraviolet–visible region-near infrared (UV–vis–NIR) spectrophotometer was used to determine the reflection (*R*%), transmittance (*T*%), and absorption (*A*) measurements of reference (nonantibacterial) borosilicate glass, antibacterial glass, and glass ceramics. This device is a dual-monochromator, double-beamed, and computer-controlled type spectrophotometer which is used to determine reflection, transmittance, and absorption measurement values in the UV–vis–NIR of the spectrum, 185–3200 nm (nanometer) in the spectral range of 200–2500 nm when using the collector spheres. UV Winlab software program operates this device, and usage of four different methods is allowed.

Using the principle of Archimedes and water as the buoyancy liquid, densities of reference (nonantibacterial) glass, antibacterial glass, and glass ceramics at room temperature were measured with a Mettler Toledo density kit.

The thermal expansion coefficients of the reference (nonantibacterial) glass, antibacterial glass, and glass ceramics were determined with a dilatometer (Netzsch DIL 402 PC).

A Nanoindenter (M1, Nanovea) was used to measure the hardness values of the samples. The indentations were performed to a maximum load of 300 mN at loading and unloading rates of 600 mN/mN. The indentation was performed by the Berkovich tip calibrated on fused silica, and 10 indents were performed on each samples.

## 3 Results and Discussion

### 3.1 Differential Thermal Analysis of the Sample

**Figure 1:**
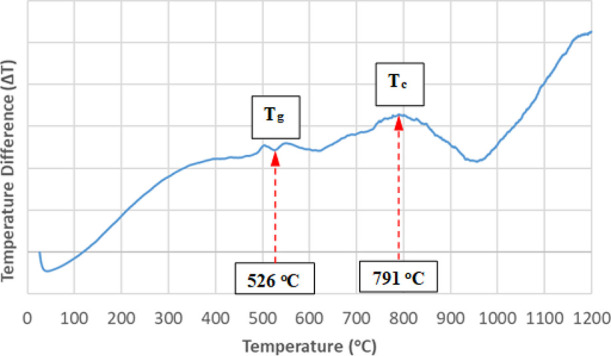
DTA curve of the borosilicate glass doped with 5.0% ZnO.

DTA was applied to determine the glass transition and crystallization temperature of borosilicate glass doped with 5.0% ZnO in the air medium at a heating rate of 10 °C/min. As can be seen from Figure 1, the glass transition and the crystallization temperatures of borosilicate glass doped with 5.0% ZnO were determined as 526 and 791 °C, respectively. It is obvious from the crystallization peak at the DTA graph that the borosilicate glass doped with 5.0% ZnO has a suitable composition for the glass ceramic production. Based on the DTA results and the preliminary experimental studies, the heat treatment process for the borosilicate glass doped with 5.0% ZnO was determined as 800 °C and 5 h at a heating rate of 10 °C/min in a muffle type furnace. After the heat treatment process, the obtained sample was cooled to room temperature slowly in the furnace.

### 3.2 Antibacterial Test Results of the Obtained Samples

**Figure 2:**
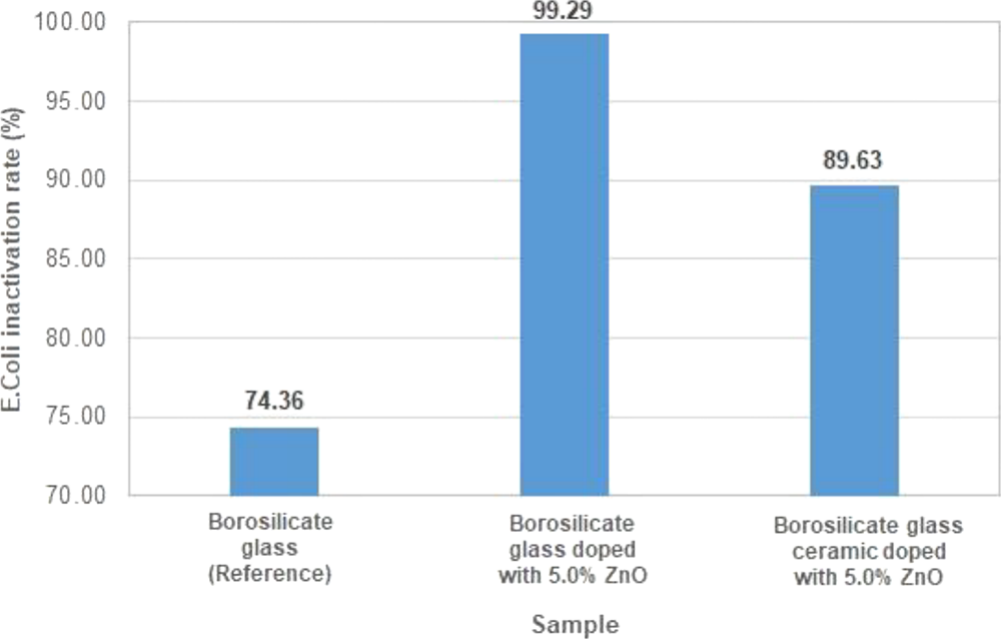
Inactivation rate of *E. coli* for borosilicate glass and glass ceramic doped with 5.0% ZnO.

Antibacterial test results for reference borosilicate glass, borosilicate glass doped with 5.0 ZnO%, and glass ceramic samples doped with 5.0 ZnO% are shown in Figure 2. Although the antibacterial activity of borosilicate glass doped with 5.0 ZnO% was at the desired level, the antibacterial activity of borosilicate glass ceramic doped with 5.0 ZnO% was below the desired level due to material structural change. The reason of that is the dissolution of zinc ions from the glass–ceramic material is slower than those of the glass sample doped with 5.0% ZnO because of the crystalline structure of the glass–ceramic sample.^44^ This means that it is difficult to release of zinc ions from the crystalline structure compared with the amorphous glass structure. Therefore, antibacterial properties of the borosilicate glass doped with ZnO are higher than those of the borosilicate glass ceramic sample doped with ZnO.

There are antibacterial borosilicate glass and glass ceramics which have high antibacterial activity against *E. coli* in the literature; however, expensive raw materials such as silver and titanium were used as an antibacterial agent in those studies.^21,45−47^ Therefore, the obtained samples in our study provide an important advantage in terms of raw material cost.

### 3.3 TCLP Test Results of the Antibacterial Samples

The TLCP tests were performed on the obtained samples to be able to determine their toxicity. The released amounts of zinc ions from the borosilicate glass doped with 5.0% ZnO and borosilicate glass ceramic doped with 5.0% ZnO were detected as 18.61 and 21.86 ppm, respectively. The amount of zinc release from the samples was lower than the maximum zinc levels that should be taken in terms of human health on the basis of age and gender according to US EPA limits.^48^ TCLP results indicated that the toxic values of the borosilicate glass and glass ceramic samples doped with 5.0% ZnO were found to be harmless to human health.

### 3.4 Characterization of the Antibacterial Glass and Glass Ceramic Sample

**Figure 3:**
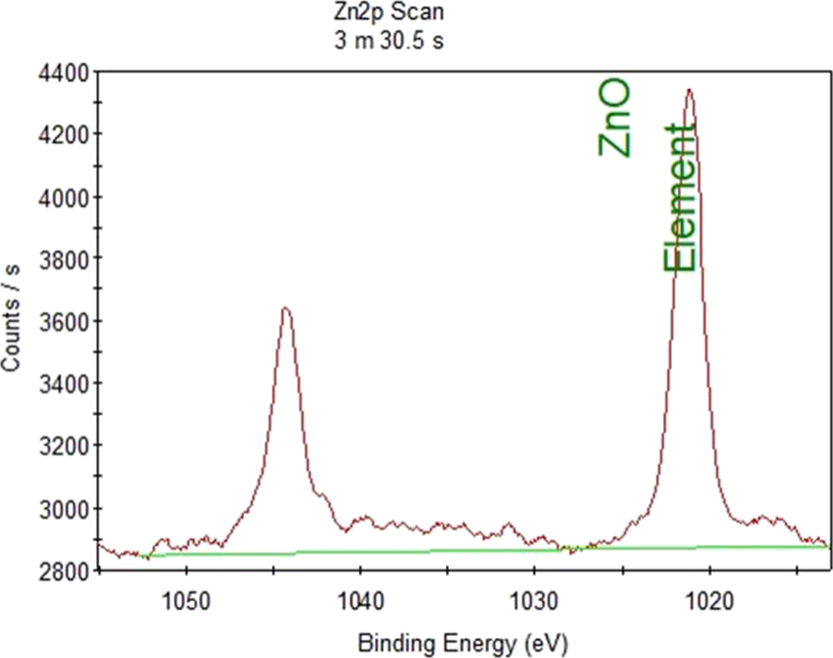
XPS analysis of the borosilicate glass sample doped with 5.0% ZnO.

**Figure 4:**
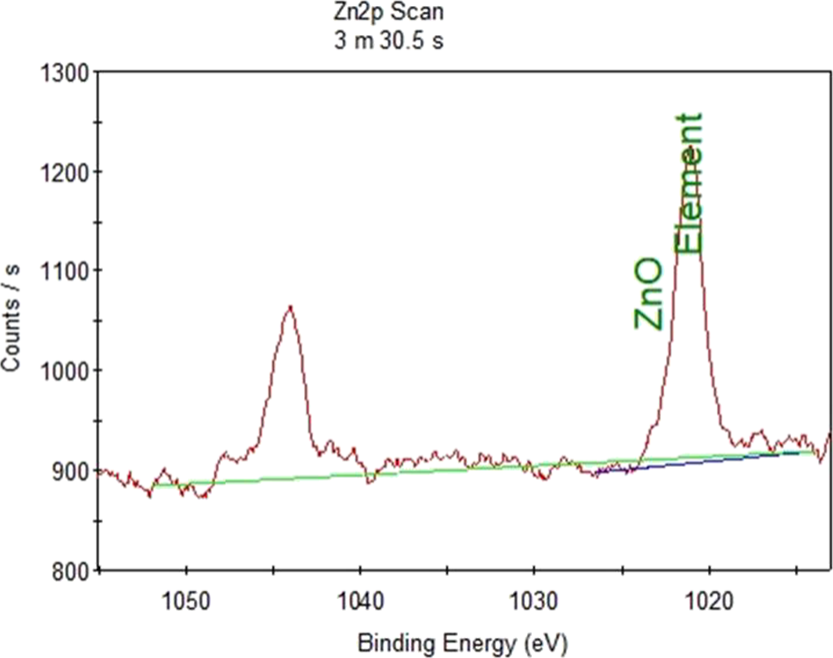
XPS analysis of the borosilicate glass ceramic sample doped with 5.0% ZnO.

XPS analysis was done in order to determine the energy levels of zinc oxide for antibacterial borosilicate glass and borosilicate glass ceramic samples. Zinc oxide covers the range of 1020–1050 eV, and the expanded graph of this region is shown in Figure 3 for antibacterial borosilicate glass sample and Figure 4 for the antibacterial borosilicate glass ceramic sample. XPS analysis results indicated that zinc oxide successfully participated in the structure of borosilicate glass and glass ceramic doped with 5% ZnO. This result also confirmed that the antibacterial property of the glass sample since zinc oxide was detected on the surface of the samples.

**Figure 5:**
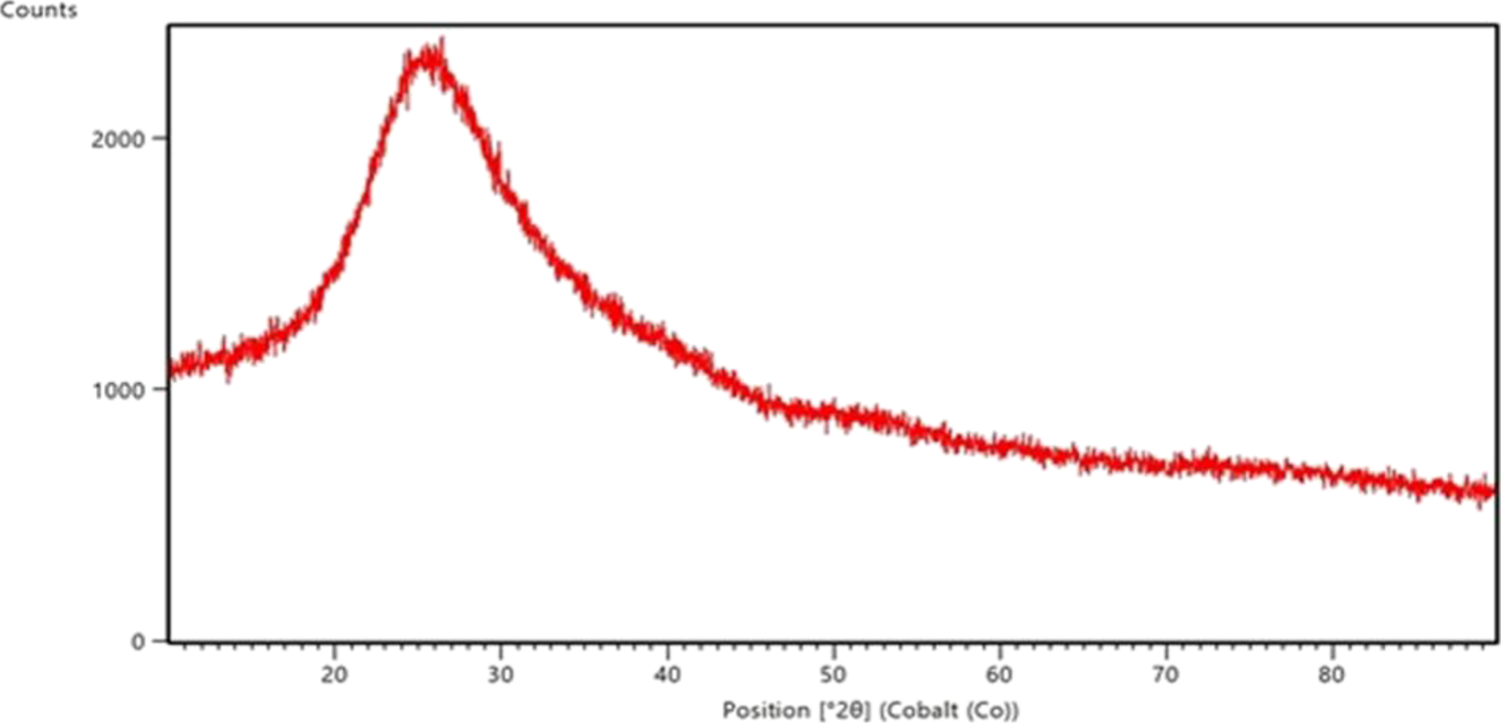
XRD pattern for borosilicate glass doped with 5.0% ZnO.

**Figure 6:**
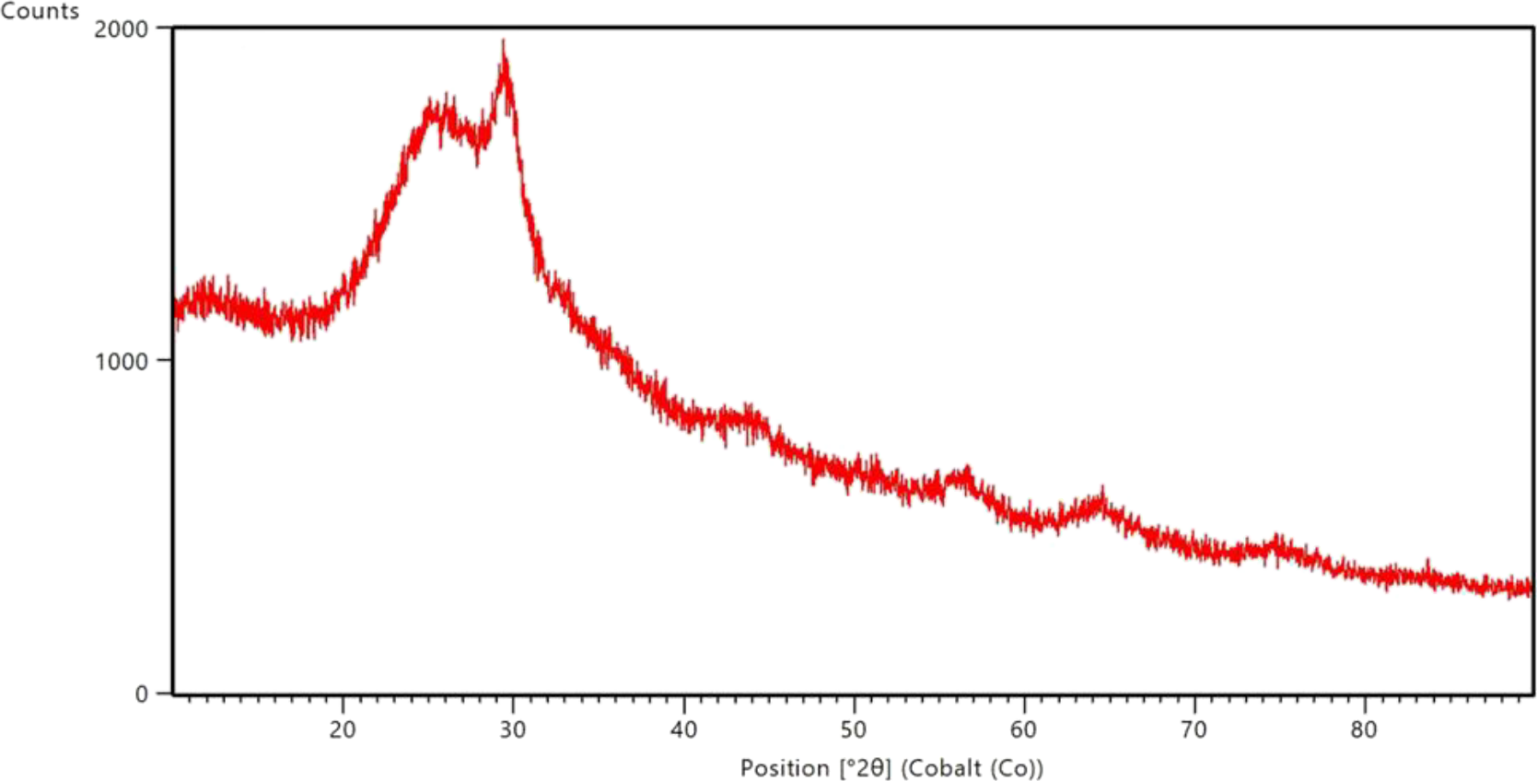
XRD pattern for borosilicate glass ceramic doped with 5.0% ZnO.

From Figure 5, it was seen that the XRD pattern had a broad diffraction peak between 20 and 30°, and there was no peak associated with any crystalline phase.^49^ Therefore, the borosilicate glass doped with 5.0% ZnO had an amorphous structure. From Figure 6, it was seen that the crystalline structure was observed as natural quartz (sharp lines) at nearly 30° for the borosilicate glass ceramic doped with 5.0% ZnO.^49^ Therefore, it was proven that the borosilicate glass doped with 5.0% ZnO transformed into a glass–ceramic structure. However, there was still amorphous structure in the borosilicate glass–ceramic doped with 5.0% ZnO.

**Figure 7:**
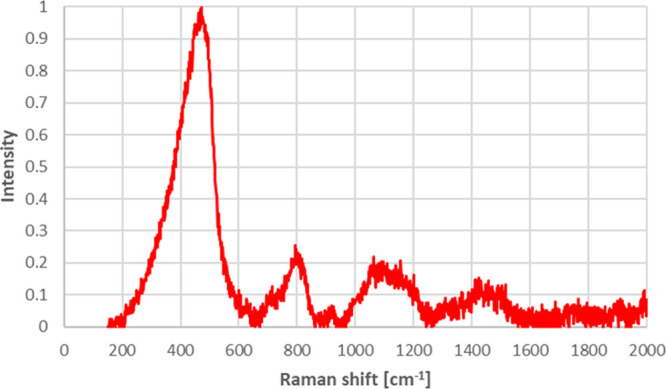
Raman spectra for borosilicate glass doped with 5.0% ZnO.

**Figure 8:**
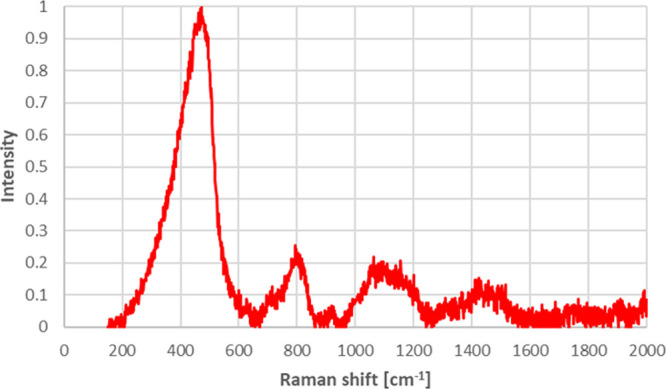
Raman spectra for borosilicate glass ceramic doped with 5.0% ZnO.

As seen from Figures 7 and 8, the Raman spectra of borosilicate glass doped with 5.0% ZnO and borosilicate glass ceramic doped with 5.0% ZnO were similar to each other. Prominent peaks nearly at 470 cm^–1^ belong to SiO_2_, prominent peaks nearly at 800 cm^–1^ belong to B_2_O_3_, and prominent peaks nearly at 1100 cm^–1^ belong to zinc oxide.^50−52^ Raman analysis results also confirmed the XPS results by indicating that zinc oxide was successfully incorporated into the borosilicate glass doped with 5.0% ZnO and borosilicate glass ceramic doped with 5.0% ZnO.

**Figure 9:**
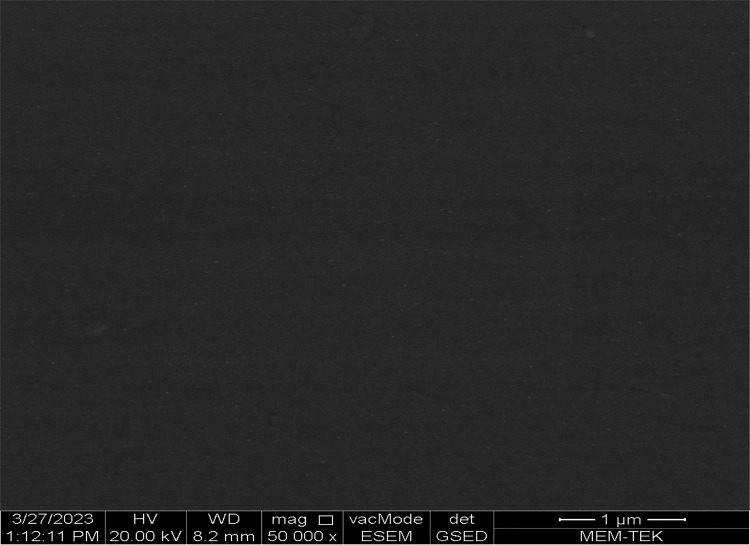
SEM image of borosilicate glass doped with 5.0% ZnO.

**Figure 10:**
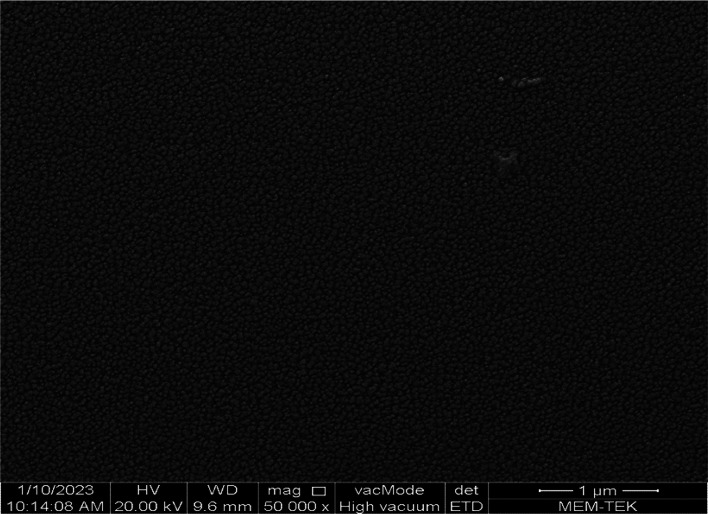
SEM image of borosilicate glass ceramic doped with 5.0% ZnO.

**Figure 11:**
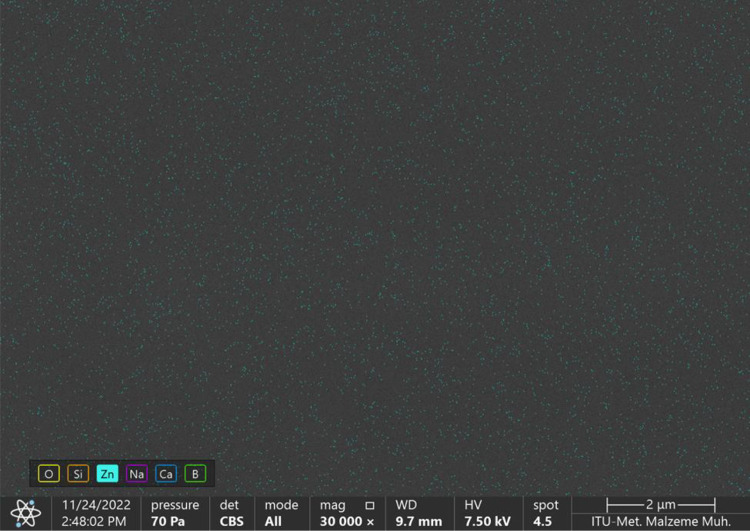
Zinc distribution of borosilicate glass ceramic doped with 5.0% ZnO on the surface.

SEM analysis was also performed on the antibacterial borosilicate glass and glass ceramic samples to investigate their crystalline morphology. From Figure 9, it was seen that there is no crystalline phase in the borosilicate glass sample doped with 5.0% ZnO. On the other hand, from Figure 10, it was clearly seen that the borosilicate glass–ceramic doped with 5.0% ZnO has spherical crystalline structures with an average diameter of 60 nm. The spherilutic crystallites in the nanosized were homogeneously dispersed on the surface of the sample as observed by SEM. In addition to nanosized crystallites, there was still amorphous structure in the sample. Furthermore, elemental mapping was also performed on the surface of the glass ceramic sample through the SEM observations. The presence of zinc on the surface of the borosilicate glass ceramic doped with 5.0% ZnO was also observed from Figure 11 through the elemental mapping. These results showed that the borosilicate glass doped with 5.0% ZnO could be transformed to the glass ceramic structure.

Moreover, additional tests were performed on the antibacterial glass and glass ceramic samples to be able to compare physical, thermal, and optical properties of the reference glass (nonantibacterial borosilicate glass) with those of the antibacterial glass and glass ceramic (5% ZnO). Table 1 shows the obtained results. The physical, thermal, and optical properties of antibacterial glass and the reference borosilicate glass (nonantibacterial) were found to be close to each other except the thermal expansion coefficient. An improvement was observed in the thermal expansion coefficient of borosilicate glass and glass ceramic samples doped with 5.0% ZnO. In addition to the antibacterial property, the improved thermal expansion coefficient makes the borosilicate glass and glass–ceramic samples valuable materials for the kitchenware usage.

**Table 1:** Physical and Optical Test Results of Borosilicate Glass and Glass Ceramic Doped with 5.0% ZnO

	reference	antibacterial borosilicate glass	antibacterial borosilicate glass ceramic
thermal expansion coefficient (10^–7^/°C)	38.0	36.2	35.1
density (g/cm^3^)	2.247	2.304	2.312
refractive index	1.4755	1.4840	1.4815
color parameters (std. 3 mm)			
dominant wavelength (nm)	487.2	571.4	571.1
brightness (%)	91.0	89.9	88.9
purity (%)	0.2	0.3	0.4

The Vickers’ hardness values of the borosilicate glass and borosilicate glass ceramic samples were measured as 539 ± 20 and 662 ± 30 kg/mm^2^, respectively. The hardness value of borosilicate glass ceramic doped with 5.0% ZnO was higher than that of borosilicate glass doped with 5.0% ZnO as it was expected. Based on the SEM investigation, the nanocrystalline structure of the borosilicate glass ceramic sample was observed. The factors regulating physical, mechanical, and chemical properties are crystalline phase, crystallization degree, the size of the crystallites, and homogeneity of crystal size. Therefore, hardness and the thermal expansion coefficient of the borosilicate glass ceramic sample were better than those of the amorphous borosilicate glass sample.

## 4 Conclusions

In this study, glass and glass ceramic with antibacterial properties derived from the borosilicate glass composition were obtained by adding zinc oxide to the batch. DTA was performed to see the ability of the borosilicate glass composition to transform into a glass ceramic structure and crystalline structures observed by XRD, and SEM analysis confirmed the glass ceramic structure. According to TCLP results, the migration values of zinc oxide from the borosilicate glass and glass ceramic doped with 5.0% ZnO were lower than toxic values. The physical, thermal, and optical properties of antibacterial samples and reference glass were nearly the same. However, hardness and thermal expansion coefficient values of the borosilicate glass ceramic sample were better than those of borosilicate glass because of the nanocrystalline structure of the borosilicate glass ceramic sample. The importance of producing vials for vaccines in the COVID-19 pandemic period and the market size of vials has been growing. Therefore, it was thought that the obtained antibacterial borosilicate glass may be a good candidate for the healthcare sector especially in pharmaceutical packaging of vaccines. Furthermore, the improved thermal expansion coefficient and the hardness values of the antibacterial borosilicate glass–ceramic sample make it suitable as an antibacterial material for kitchenware usage.
